# Genetic diversity and population structure of the Mediterranean sesame core collection with use of genome-wide SNPs developed by double digest RAD-Seq

**DOI:** 10.1371/journal.pone.0223757

**Published:** 2019-10-10

**Authors:** Merve Basak, Bulent Uzun, Engin Yol

**Affiliations:** Department of Field Crops, Faculty of Agriculture, Akdeniz University, Antalya, Turkey; National Cheng Kung University, TAIWAN

## Abstract

The Mediterranean sesame core collection contains agro-morphologically superior sesame accessions from geographically diverse regions in four continents. In the present investigation, the genetic diversity and population structure of this collection was analyzed with 5292 high-quality SNPs discovered by double-digest restriction site associated DNA (ddRAD) sequencing, a cost-effective and flexible next-generation sequencing method. The genetic distance between pairs of accessions varied from 0.023 to 0.524. The gene diversity was higher in accessions from Asia than from America, Africa, and Europe. The highest genetic differentiation was observed between accessions collected from America and Europe. Structure analysis showed the presence of three subpopulations among the sesame accessions, and only six accessions were placed in an admixture group. Phylogenetic tree and principal coordinate analysis clustered the accessions based on their countries of origin. However, no clear division was evident among the sesame accessions with regard to their continental locations. This result was supported by an AMOVA analysis, which revealed a genetic variation among continental groups of 5.53% of the total variation. The large number of SNPs clearly indicated that the Mediterranean sesame core collection is a highly diverse genetic resource. The collection can be exploited by breeders to select appropriate accessions that will provide high genetic gain in sesame improvement programs. The high-quality SNP data generated here should also be used in genome-wide association studies to explore qualitative trait loci and SNPs related to economically and agronomically important traits in sesame.

## Introduction

Sesame, *Sesamum indicum* L. (2n = 26), is an ancient oilseed crop belonging to the genus *Sesamum*, family Pedaliaceae, in the order Tubiflorae. Sesame have been used for oil and food for more than 4000 years [1], and it has been referred to as “the queen of the oilseeds” [2]. The crop is cultivated globally in more than 9 million ha area, mostly in tropical and subtropical regions, with annual yields of about 5.5 m t of seeds [3]. The oil content of sesame seeds is about 50–60%, which is higher than that of major oil seeds like peanut, rapeseed, soybean, and sunflower. The oil profile is rich in unsaturated fatty acids (about 80%) and low in saturated fatty acids, and mainly includes stearic (C18:0) and palmitic (C16:0) acids [4]. The oil is also highly resistant to oxidative deterioration, thanks to the presence of unique antioxidant lignans, including sesamin and sesamolin [5, 6]. Sesame has many agricultural attributes; it can grow on only soil moisture without irrigation, it can be grown in mixed stands with different crops, and it can set seed and yield well under high temperatures with low fertilizer inputs [7]. However sesame has a low yield when compared to other commercial oil crops because of its residual wild traits, including capsule shattering [8], indeterminate growth habit [9], nonsynchronous maturity, low environmental adaptability [10], and susceptibly to phyllody disease [11] Further yield limitations arise because of agricultural issues, such as the use of mixed local landraces, low harvest index, inadequate cultural practices, poor crop rotations, and a lack of advanced breeding lines and high-yielding cultivars [7]. The understanding and exploitation of plant genetic diversity are therefore highly important in genetic and breeding research in sesame.

Several agro-morphological characterization studies have been conducted on various genetic resources of sesame [12–15] to identify remarkable traits and genotypes from different climatic zones. The use of molecular markers has also allowed detailed characterizations of germplasm to distinguish sesame genotypes and to identify genetic diversity [16]. Different molecular markers, such as random amplified polymorphic DNA (RAPD) [17, 18], amplified fragment length polymorphism (AFLP) [19], inter-simple sequence repeats (ISSR) [20], genomic simple sequence repeats (SSR) [21], and insertions and deletions (InDels) [22], have been applied to evaluate the genetic diversity and population structure of selected sesame populations. Recently, single-nucleotide polymorphisms (SNPs) based on next-generation sequencing (NGS) have been utilized in sesame genetic diversity studies [23, 24]. SNPs have a number of advantages, as they are extremely abundant in the genome, are unbiased [24], have a wide genomic distribution [25], and are speed/cost effective with respect to the number of markers produced.

In the last decade, different types of high-throughput and cost-effective SNP detection platforms have been developed [26]. Of these, double-digest restriction site associated DNA (ddRAD) sequencing [27] is one of the most important and has been widely used simultaneously for SNP discovery and genotyping in next-generation biological research. The ddRAD approach produces high-density genome-wide SNPs after suitable restriction enzyme digestion, while reducing the complexity of the studied genome, and provides data that can be efficiently analyzed without the requirement for a reference genome sequence [27]. Basically, this method involves the following consecutive steps in library preparation: (i) low- and high-frequency cuts to digest the DNA, (ii) ligation of a barcoded adapter to one restriction site and a common adapter to the other, (iii) pooling, (iv) size selection, (v) library enrichment, and (vi) introduction of a second barcode in the form of an Illumina index to increase multiplexing [28]. When compared to restriction site associated DNA sequencing (RAD-Seq) [29], the ddRAD method uses double restriction enzyme digestion, which reduces the library preparation cost, and it provides a precise size selection, making it a powerful and economical SNP diagnostic technique for genotyping [30]. The elimination of random sharing in ddRAD library preparation also reduces the number of datasets containing high percentages of missing data, which is one of the major drawbacks of the genotyping-by-sequencing (GBS) approach [31]. The data generated by ddRAD have been used in various crops for linkage mapping [32], QTL studies [33], and GWAS [34]. Studies have also been conducted with ddRAD technology to assess the genetic diversity of different plant resources, such as apple [35], orchid [36], and onion [37]. However, ddRAD technology has not yet been used for genomic characterization of sesame germplasm, including accessions from different origins.

The comprehensive and accurate characterization of germplasm and, therefore, the utilization of this germplasm in plant breeding research is limited by the large amount of genetic resources. An efficient selection of these genetic resources can be obtained using the core collection concept, which allows the use of a limited set of genotypes to represent the whole collection with a minimum of repetitiveness [38]. In sesame, the Mediterranean sesame core collection has been selected using a principal component score strategy from a worldwide sesame collection [15] that includes 345 genotypes representing 29 sesame-growing countries. This core collection contains high-yielding genotypes as well as those with commercially important traits, such as different seed colors, early flowering, adaptability [15], high oil content [39], and phyllody resistance [40]. Evaluation of the genetic variation and differentiation of genotypes in this collection at the genomic level will allow a more effective use of these valuable genetic resources to exploit new alleles that could be exploited in breeding programs to enhance the productivity of sesame. The aim of the present study was therefore to apply the ddRAD approach to identify SNPs at a genome-wide scale and to understand the genetic diversity and population structure in the Mediterranean sesame core collection.

## Materials and methods

### Plant materials

The Mediterranean sesame core collection consists of 103 accessions [15], and 95 available accessions were used in the present study, representing genetic material from 21 geographic regions spread over four different continents (S1 Table). The highest number of accessions in the collection is from Turkey (15), followed by those from Iran (11), the USA (10), Russia (9), and Pakistan (9). The continents of Asia, Europe, America, and Africa were represented by 61.0%, 17.9%, 16.8%, and 4.2% of the accessions, respectively.

### DNA extraction, library construction, and SNP calling

Each accession was grown in pots, and the leaves were harvested from 3 week-old plants. DNA was extracted using the CTAB method [41] with minor modifications in which extra chloroform-isoamyl alcohol and 70% ethanol cleaning steps were incorporated. The quality and quantity of DNA was checked by running it on a 1% agarose gel and the amount was normalized to 100 ng/μL by comparing lambda DNA standard before genotyping. Several DNA samples were randomly selected and tested with *Msp*I to validate the restriction enzyme digestion.

A reduced representative genomic library was prepared before subjecting each sesame accession to Illumina 150bp PE sequencing. A modified version of the ddRAD method [27] was followed for genomic library construction. Briefly, a six-base cutter *Vsp*I restriction enzyme was used instead of the *EcoRI* restriction enzyme employed in the original ddRAD protocol. Digestion products were cleaned with Ampure XP beads (Beckman Coulter Genomics) followed by P1 and P2 adapter ligation using T4 ligase buffer. The 3’ end of theP1 adapter was also modified to complement the overhanging *Vsp*I restriction cut site. Following ligation, each reaction was subjected to PCR amplification (15 cycles) using genotype specific indexed PCR primers. The PCR products were then checked on an agarose gel and pooled in equal concentration. A final step, the pooled products were size selected (400-500bp). The ddRAD sequencing data have been deposited in the National Center for Biotechnology Information (NCBI) Sequence-Read Archive (SRA) database with the accession number of PRJNA560319.

The raw reads were demultiplexed with Je (v1.2) [42] and organized into individual genotype specific fastq sanger files. The generated single files for each genotype were handled with fastp [43] for a quality check. Reads with a Phred quality score less than 15 out of 40 and containing *Vsp*I and *Msp*I sequences were trimmed. The processed sequences were then aligned to the sesame reference genome “Zhongzhi13 V2.0” [44] using Bowtie2 software [45] with the default parameters. The SNP calling tools were operated under the Galaxy (www.usegalaxy.org) software framework. Genotype specific individual BAM (binary sequence alignment file format) files were subjected to variant calling using freebayes (Galaxy Version 1.1.0.46–0) [46], with the simple diploid calling with filtering and coverage values of 20X. Insertions and deletions (In/Del) were discarded from each vcf files with VCFfilter (Galaxy Version 1.0.0). Each genotype specific vcf files including only SNP variants were then merged with the VCFgenotypes (Galaxy Version 1.0.0) to form a single data file. Combined vcf file resultant in 56648 SNP variants were converted to BED (Browser Extensible Data) file format for indication of variant genomic regions. Following this step, each BAM files were rerun on freebayes with newly created BED file with 6X coverage filtering option to identify reliable SNP alleles for each genotype. Resultant individual vcf files were combined in a single file with VCFgenotypes (Galaxy Version 1.0.0). In the last point, the merged SNPs were filtered with Tassel V5.2.52 [47] for further analysis using the parameters Minor Allele Frequency (MAF) >0.05 and proportion of missing data <3%. This data file was used in further analysis.

### Genetic diversity

Before conducting the genetic and population analyses, the SNP-containing file (.vcf) were converted to the related software input file formats by PGD Spider v2.1.1.5 [48]. Genetic diversity analysis was conducted using GenAlex program V6.5 [41]. The number of alleles, proportions of allele occurrence, and distribution of SNPs in each chromosome were calculated with Tassel v5.2.52 [47]. The population structure was determined using the Bayesian clustering approach implemented in STRUCTURE V2.3.4 [49], employing the admixture model with correlated allele frequencies. Twenty independent runs were performed by setting the number of populations (*K*) from 2 to 8 to identify the optimal number of populations present within the95 accessions. The following parameters were used: a burn-in period of 10000 and a Markov Chain Monte Carlo replication of 10000. The best *K*-value (the number of subpopulations) was estimated with the use of the *ad-ho*c statistic ΔK [50] via the online software Structure Harvester V0.6.94 (http://taylor0.biology.ucla.edu/structureHarvester) [51]. Each sesame accession was then assigned to a cluster (Q) based on a probability determined by STRUCTURE V2.3.4, which provided clustering for the accessions. The cut-off probability for assignment to a cluster was 0.50 for the clusters. The population structure bar plots were generated with the STRUCTUREPLOT (http://btismysore.in/strplot) online tool [52], with genotype labels and the created plot ordered by Q-value.

A principal coordinate analysis (PCoA) was performed with the PAST software V3.23 [53]. The phylogenetic tree was constructed with Tassel v5.2.52 using the un weighted pair group method with arithmetic mean (UPGMA) [54] clustering method and modified in FigTree v1.4.4 (http://tree.bio.ed.ac.uk/software/figtree). The software Arlequin V3.5 [55] was used to identify the pairwise genetic distance (F_ST_) for the subpopulations and to calculate the genetic variation between and within geographical groups with analysis of molecular variance (AMOVA). An F_ST_ value of 0 indicated no genetic divergence within the subpopulations and a value of 1 indicated complete extreme division. Populations were considered to have little differentiation when F_ST_ ≤ 0.05, moderate differentiation when 0.05 < F_ST_ ≤ 0.15, strong differentiation when 0.15 < F_ST_ ≤ 0.25, and very strong differentiation when F_ST_> 0.25 [56].

## Results

### Genotyping

A total of 349.86 M raw sequence reads were acquired from 150 bp paired-end sequencing on the Illumina Hiseq platform after quality filtering. The collection was presented by a mean of 3.68 M reads and 38% guanine-cytosine (GC) content per accession (S1 Fig). The highest and lowest reads were 1.55 and 6.33 M for the accessions ACS15 and ACS87, respectively. The mean of the ddRAD reads mapped to the *Sesamum indicum* L. reference genome was 81.89%. A total of 56648 unique SNPs were initially called from the accessions using a variant calling pipeline. In total, 5292 polymorphic SNP markers were selected (Table 1) for further analysis after high quality filtering, which allows only 3 missing data points in each locus and MAF > 0.05. The 5241 filtered SNPs were mapped onto 13 sesame chromosomes, and the remaining 51 SNPs were unmapped. The highest number of SNPs was detected on Chromosome 3 (653 SNPs), whereas the lowest number of SNPs was found on Chromosome 7 (251 SNPs), with an overall mean of 403 SNPs per chromosome. The average SNP density was 46 kb, with the lowest and highest SNP densities observed on chromosomes 12 and 7, respectively. The maximum and minimum SNP frequency occurred as C/T and A/C, respectively (Table 2). The proportion of transitions (3001 allelic sites, 56.71%) was greater than transversions (2291 allelic sites, 43.29%) (Table 2).

**Table 1:** Distribution SNPs in of each chromosome.

Chromosome	Initial number of SNPs	The filtered number of SNPs	Average map length per SNP (kb)
Entire genome	56648	5292	49
Chr 1	5094	454	45
Chr 2	3606	333	53
Chr 3	6496	658	40
Chr 4	3783	353	59
Chr 5	3253	260	64
Chr 6	5792	490	53
Chr 7	2946	251	67
Chr 8	5552	505	52
Chr 9	5253	561	41
Chr 10	4181	327	60
Chr 11	3157	304	47
Chr 12	4156	468	35
Chr 13	2922	277	60
Scaffolds	457	51	-

**Table 2:** SNP statistics in filtered data indicating number of allele and frequency of allele occurrence.

Allele*	Number of allele	Proportion of allele occurrence
C:T	799	15.10
A:G	777	14.68
G:A	747	14.12
T:C	678	12.81
T:A	331	6.26
C:G	320	6.04
A:T	297	5.61
T:G	290	5.48
G:C	279	5.27
C:A	266	5.03
G:T	260	4.91
A:C	248	4.69

*The first and second letters are reference and alternate alleles

### Population genetics and diversity

Genetic diversity analysis for the collection and the continental subgroups was conducted with GenAlex program V6.5 [57]. The means of the effective and observed allele numbers for the collection were recorded as 1.89 and 1.47, respectively, (S2 Table). The highest number of different alleles was observed in accessions from Asia. The expected heterozygosity (Nei’s gene diversity) was the highest in the Asian group, with a value of 0.31, followed by the African and American groups. The general mean of expected heterozygosity was 0.283. Similarly, the Shannon diversity index was highest in the Asian group. The percentages of polymorphic loci per group varied from 70.56 to 99.66, with a mean value of 87.80. The genetic distance between the 95 sesame accessions was also calculated with the distance matrix option of TASSEL V5.2.52 (S3 Table) and ranged from 0.023 to 0.5243. The highest and smallest genetic distances were observed between accessions ACS 65–ACS 87 and ACS 304–ACS 329, respectively.

**Fig 1:**
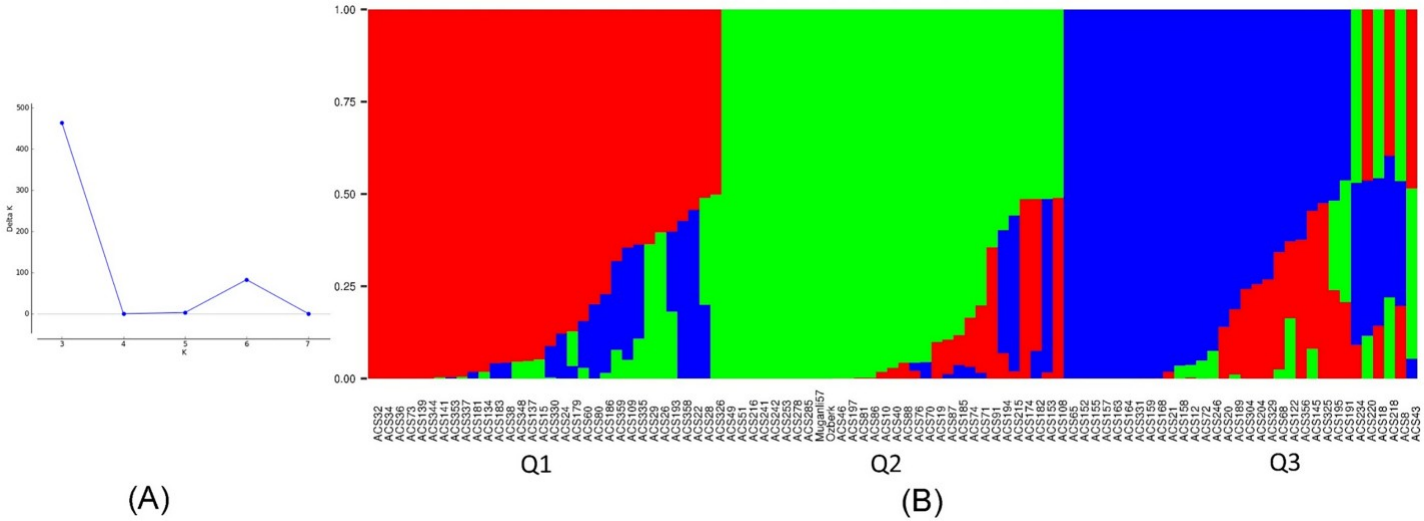
(A) Delta K values for different numbers of populations assumed (K) in the STRUCTURE analysis. (B) Classification of 95 sesame accessions into three populations (K = 3) using STRUCTURE v2.3.4. Each accession is represented by a single row, which is partitioned into colored segments in proportion to the estimated membership in the three subpopulations. Numbers on the y-axis show the subgroup membership, and the x-axis shows the different accession.

The 5292 genome-wide SNPs were used for analysis of the population structure of the Mediterranean sesame core collection. The software STRUCTURE V2.3.4 determined the hierarchical population structure with by setting the number of subpopulations (*K*) from 2 to 8 and conducting twenty runs for each *K*-value. The results obtained from STRUCTURE were assessed via Structure Harvester [42] to identify the optimum *K* value. The largest delta *K* was observed at *K* = 3, suggesting the presence of three main clusters (Q1, Q 2 and Q3) in the sesame panel (Fig 1). The accessions were considered to be part of a group when the probability of membership threshold was 0.50. Q1 had 32 accessions from Asia (21) and America (11), Q2 was composed of 31 accessions from all continents, and Q3 had 25 accessions originating mainly from Asia (16). The remaining 7 genotypes (ACS 8, ACS 18, ACS 43, ACS 191, ACS 218, ACS 220, and ACS 234) were classified into an admixture group.

Genetic differences among the continental groups were assessed with AMOVA. The results indicated that 5.53% of the variation was among the continental groups, whereas 66.28% was among individuals within the continental groups (Table 3). Of the remaining variation, 28.18% was within individuals. Analysis of the pairwise genetic differentiation among the four continental groups showed that the highest value of F_ST_ (0.15, P<0.001) was between the American and European continental groups. The corresponding F_ST_ values between the continental groups were moderate for Asia vs. Europe (0.09, P<0.001) and lower for Asia vs. Africa (0.05) and Asia vs. America (0.04) (Table 4).

**Table 3:** Analysis of molecular variance (AMOVA) results for global FST statistics.

Source of variation	d.f.	Sum of squares	Variance components	Percentage of variation
Among continental groups	3	8867.774	45.58928	5.53
Amongindividuals within continental groups	91	120512.053	546.05677	66.28
Withinindividuals	95	22058.500	232.19474	28.18
Total	189	151438.326	823.84078	

**Table 4:** Pairwise Fst values between continentals which includes accessions from same origin.

Asia			Africa		America
Africa	America	Europe	America	Europe	Europe
0.05	0.04	0.09***	0.07	0.10	0.15***

*** Significant at P ≤ 0.001 (110 permutations).

**Fig 2:**
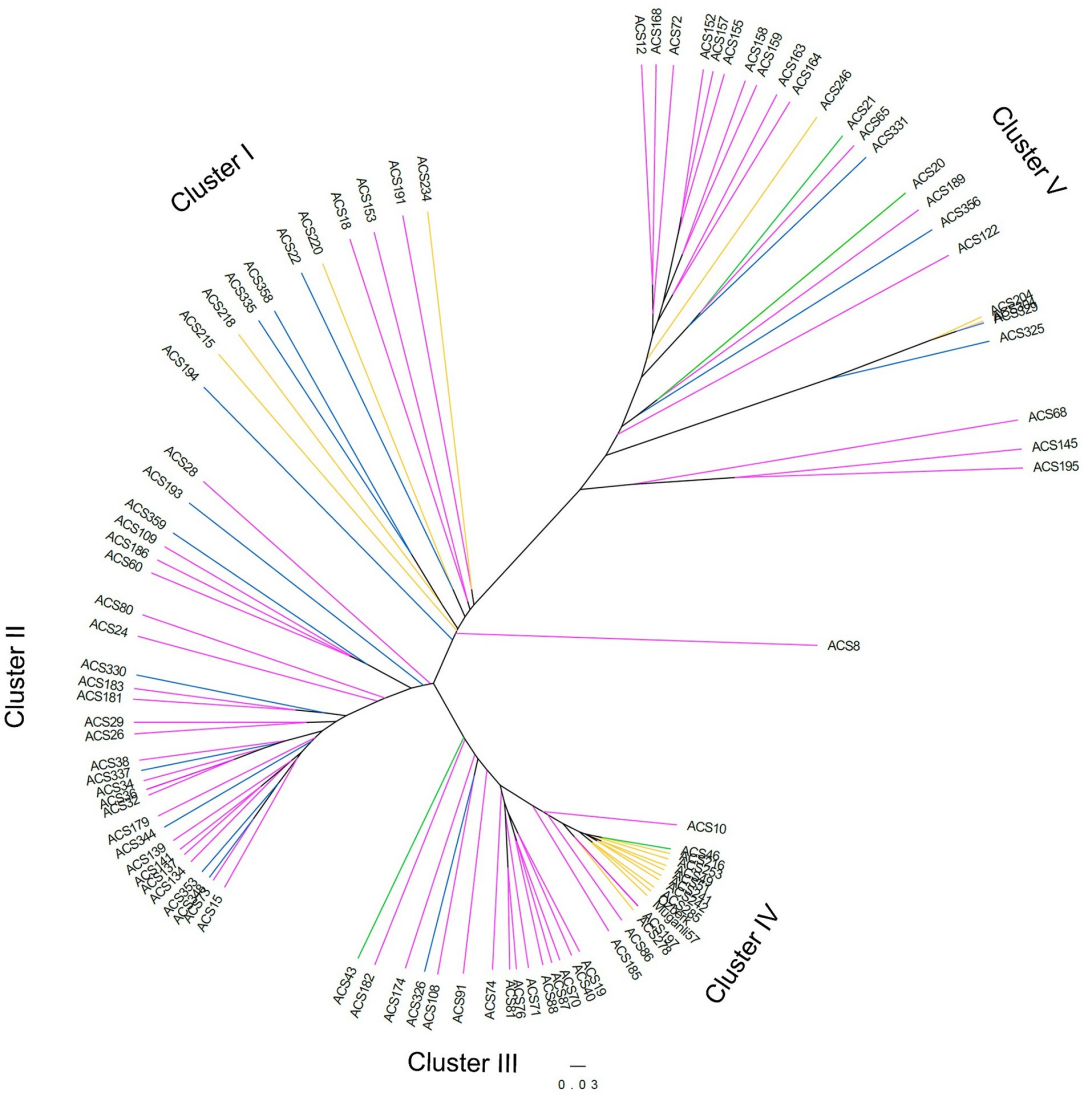
UPGMA based dendrogram generated using 5292 SNPs and 95 sesame accessions. The colors of pink, green, blue and yellow represent continental origins of accessions. Pink is Asia; Green is Africa; Blue is America; Yellow is Europe.

A phylogenetic tree consisting of 95 sesame accessions was constructed based on the UPGMA clustering method with 5292 high-quality SNPs obtained from ddRAD (Fig 2). The sesame accessions were divided into five clusters according to the continents and countries of origin. The clustered group I contained 11 accessions originating from three different continents, with no clear majority from any single continent. Clustered group II had the highest number of accessions (28) from Asia and America. Clustered groups III and IV consisted of 15 and 22 accessions, respectively, originating from all the studied continent groups, but most of the accessions were from Asia. Clustered group V included 15 accessions, mostly originating from Europe.

**Fig 3:**
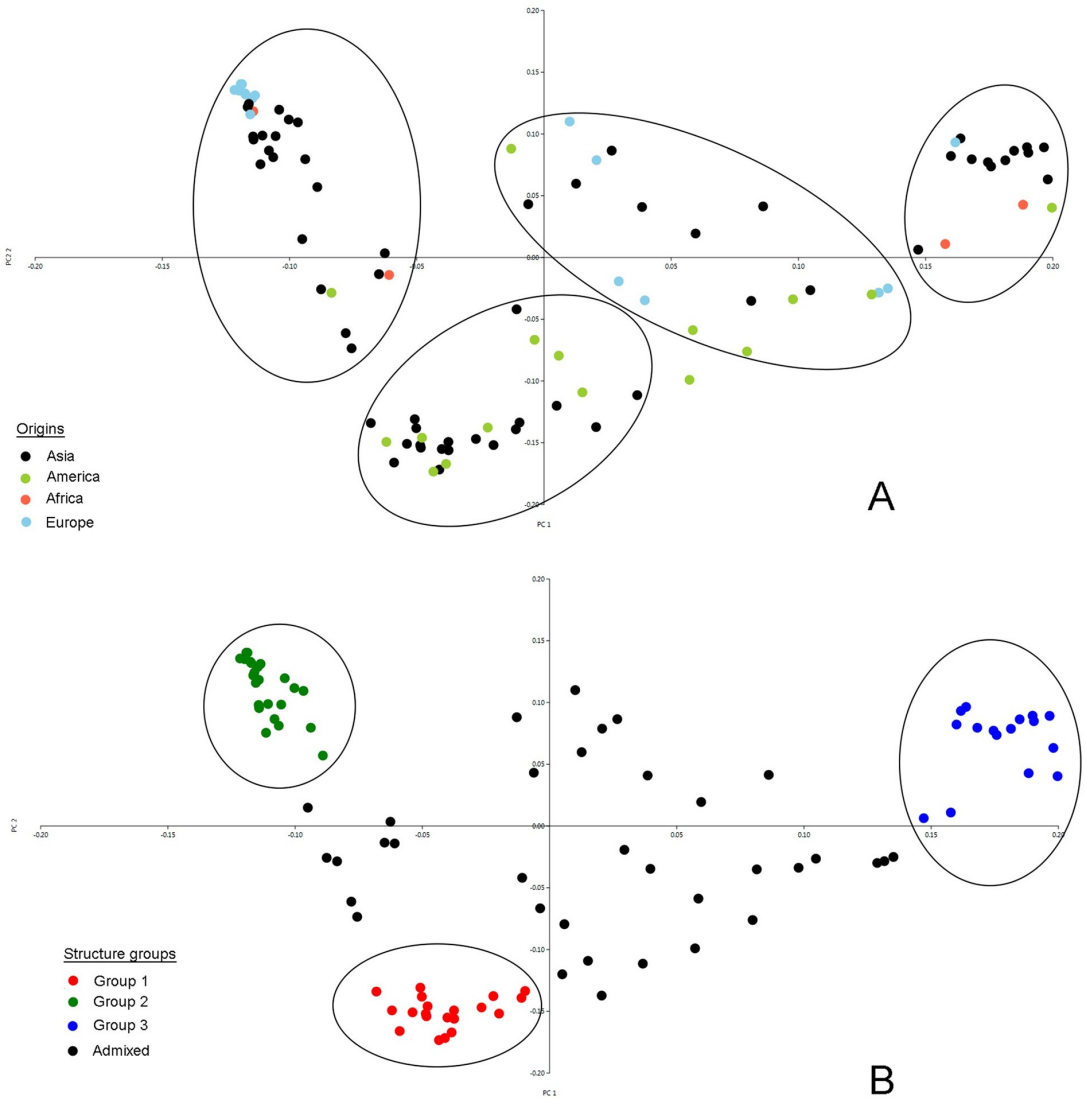
(A) Principal coordinate analysis (PCoA) of 95 sesame accessions based on 5292 SNP markers. Colors reflect continental origin of accessions. (B) PCoA separated by group membership in STRUCTURE at K = 3. Circles have >80% membership colored red, green and blue circles; black circles are admixed at rate of <80%.

Principal coordinate analysis (PCoA) indicated the existence of four subgroups among the95 accessions of our study (Fig 3). The first and second coordinate explained 27.3% and 11.1% of the variation, respectively. The sesame panel was also divided into three clusters corresponding to three subgroups (Group I, Group II, and Group III) ascertained using STRUCTURE with *K* = 3. All groups had accessions from Asia, and Group I, Group II, and Group III consisted of 20, 24, and 15 accessions, respectively, from different geographic regions and continents. The remaining accessions were clustered as an admixture, based on structure analysis.

## Discussion

Revealing the population structure and diversity of a collection is the best way to achieve efficient management of crop genetic resources to improve breeding programs and to understand the phylogenetic relations of accessions. The present investigation used genome-wide SNP analysis to investigate the diversity and population structure of the Mediterranean sesame core collection consisting of 95 accessions from 21 geographically different regions. A total of about 350 M raw sequence reads were acquired from the next-generation sequencing platform, and the number of reads varied between 1.55 and 6.33 M for the accessions in the collection (S1 Fig). This fluctuation in number of reads per accession may arise from the short read length, problems in PCR, sequencing errors, and the depth of coverage [58]. The genetic resources examined in this study were characterized using 5292 high-quality SNPs detected with the ddRAD approach. The SNPs were evenly distributed throughout the genome, showing that ddRAD is an efficient next-generation sequencing method and should be widely applied to a range of biological problems in sesame. The number of molecular markers reported in the present study is higher than the numbers reported in previous studies that investigated the geographical and evolutionary relationships of sesame accessions. For example, the genetic diversity of 58 accessions from over 21 sesame-growing countries were previously characterized with 30 random amplified polymorphic DNA (RAPD) markers [59], while 96 accessions collected from different parts of world were analyzed using 21 amplified fragment length polymorphism (AFLP) markers [60]; 404 indigenous landraces from a sesame core collection in China were evaluated by 11 sequence-related amplified polymorphism (SRAP) and 3 simple sequence repeat (SSR) markers [61]; and 96 sesame accessions from 22 different countries were genotyped using 33 SSR markers [62]. A few studies have been conducted to identify the genetic relationships of sesame accessions based on SNPs. For instance, Wei et al. [23] and Cui et al. [24] identified higher numbers of SNPs than in the present study. Differences in the sequencing approaches and in the numbers of accessions might have led to these differences in the numbers of captured SNPs, because Wei et al. [23] sequenced 366 sesame accessions using specific locus amplified fragment sequencing (SLAF-seq), while Cui et al. [24] sequenced 705 accessions using whole genome sequencing. However, the distribution of SNPs was not homogenous in these studies. The differences in recombination rates [63] and mutations [64] and the possible selection pressures on chromosomes [65] might be the causes of this uneven distribution of SNPs.

The average value of gene diversity (0.28) was higher in the present study than in the earlier reports for the sesame collections analyzed with different marker types [24, 61, 66]. Conversely, the level of genetic diversity that we observed here was lower than that observed in the collections characterized using SSR markers [21, 62]. The broad range of gene diversity among collections might be a source of the differences observed in genetic resources (such as landraces, advanced breeding lines, cultivars, etc.), sampling approaches, and number of markers [62]. The type of marker is also an important factor for the identification of gene diversity; in general, SSR markers are more productive than SNPs [67]. Considering the accessions based on their continental origin, Asia was more diverse when compared to Africa, America, and Europe (Additional file 2: S1 Table). This finding was expected because the geographical origin of crops generally shows a higher genetic diversity, as reported previously for cotton [68] and *Oryza* ssp. [69]. Laurentin and Karlovsky [19] also obtained higher genetic diversity in sesame accessions collected from Asia.

The cluster dendogram based on the geographical distribution of accessions showed that most sesame accessions from the same origin did not classify properly on the basis of country of origin. Similar results were reported previously in different sesame germplasm [60, 66, 70] and in other crops, including wheat [71], finger millet [72], and sorghum [73]. The reason for this unequal distribution of sesame accessions based on the geographical origin could be seed migrations by people who carried seeds for cultivation and or who traded with other regions for centuries; these practices may have caused gene flow among the different geographical areas. Similarly, Laurentin and Karlovsky [19] found no association between genetic diversity and accession origin, and they proposed that ecological and geographical factors have not played a significant role in the evolution of sesame. The present AMOVA analysis also supported the possibility of high rates of gene flow between regions because the genetic variation among the geographical groups accounted for 5.53% of the total variation (Table 3). Cultivated sesame was domesticated in India and taken to Mesopotamia by the Early Bronze Age [74]. This history indicates that *Sesamum indicum* L. seeds were dispersed to a wide geography from one center, leading to the observed genetically similar backgrounds. A majority of the accessions of Iran origin showed a tendency to cluster together with the accessions of the neighboring country, Turkey (Fig 2). This outcome fits the hypothesis that sesame seeds were dispersed to nearby countries by human activities. These distributed sesame genetic resources were later used in further breeding studies as modern cultivars were commercialized. For example, the cultivars Muganli-57 and Ozberk-82, released in Turkey, grouped with most of the Iranian accessions in the PCoA and STRUCTURE analysis, indicating that the Mesopotamian region (which is a historical region of Western Asia) might be the origin of these cultivars. Possible associations between accessions based on geographical origin were also observed for accessions from Greece and Turkey, as most of the accessions from these countries grouped together (Fig 2). These results were supported by the STRUCTURE analysis, which indicated that most of the accessions from Iran, Iraq, Turkey, and Greece grouped to the same cluster (Q2) (Fig 1). The accessions collected from the USA separated to different groups and clusters in the PCoA and dendogram graphics, respectively. However, the accessions analyzed in the present study mostly clustered with the accessions from China. The clustering of accessions from these geographical regions into similar groups may therefore reflect historical trade routes, because no geographical connection exists between Asia and America, so no gene flow is likely between these continents. In agreement with this idea, a low level of genetic differentiation (F_ST_) was observed in the accessions collected from Asia and America in the present investigation (Table 4). The accessions from Egypt and Angola were mainly divided into different clusters, although these have been collected from Africa. The exchange of plant materials between Egypt and the Asian regions during the history of sesame cultivation might have caused gene flow between these two geographical regions. Another factor that might explain the differentiation between accessions from Angola and Egypt could be selection pressure for agronomically important traits, because Angola has a very different climate than that of northern Africa.

The Mediterranean sesame core collection has useful characteristics, such as high yield [15], high oil content [39], and high phyllody resistance [40]. The SNPs obtained from this collection could benefit future breeding and association mapping work in sesame. Our diversity analysis of this core collection revealed genetic relationships among the accessions that may be valuable for parental selection in sesame improvement research. Therefore, the identification of genetically distant accessions (such as ACS 65–ACS 87) for hybridization in sesame breeding programs has the potential to lead to the development of elite varieties. The degree of genetic relationship and differentiation among genetic resources can broaden genetic diversity can also be used to combine alleles for valuable agricultural traits [75]. For example, the accession ACS 8 from Afghanistan was extremely distinct from the groups, but it had a high seed yield [15]. This accession should be used as a parent for crosses with accessions possessing other desirable traits to obtain ideal sesame types that have high seed yield, non-shattering capsules, and phyllody resistance.

## Conclusion

The present research showed the effectiveness of ddRAD in characterizing the genetic diversity and population structure of sesame collection and also demonstrated usefulness of restriction enzymes (*Vsp*I–*Msp*I) in sesame crop to obtain high quality SNPs. The gene diversity values calculated based on the 5292 SNPs suggest the Mediterranean sesame core collection is highly genetically diverse. The collection therefore presents useful genetic data for future molecular based studies. This study also supports the idea; ecological and geographical factors less effective in the evolution of sesame.

## Supporting information

S1 Fig Total number of reads and GC content (%) per accession. (XLSX)

S1 Table Summary of the sesame accessions in the Mediterranean sesame core collection characterized in the present study. (DOCX)

S2 Table Genetic variation among four populations on diversity panel of 95 sesame accessions. (DOCX)

S3 Table Genetic distance matrix for 95 accessions based on 5292 SNPs. (XLSX)
