# Supplementary material for: Genetic diversity and population structure of the Mediterranean sesame core collection with use of genome-wide SNPs developed by double digest RAD-Seq
Source: PLoS One. 2019 Oct 10;14(10):e0223757. doi: 10.1371/journal.pone.0223757 (PMC6786593; doi:10.1371/journal.pone.0223757)
Supplement: S1 Table — (DOCX) [file pone.0223757.s002.docx]

**S1 Table.** Summary of the sesame accessions in the Mediterranean sesame core collection characterized in the present study.

| **Accession No.** | **USDA**  **Genebank Entry / Cultivar Name** | **Country of origin** | **Continent** | **Cluster (Q) membership based  on STRUCTURE at K=3*** | **Group (G) membership based on PCoA analysis^¶^** |
| --- | --- | --- | --- | --- | --- |
| ACS 8 | PI 220403 | Afghanistan | Asia | Admixture | Admixture |
| ACS 10 | PI 220544 | Afghanistan | Asia | Q2 | Group II |
| ACS 12 | PI 223532 | Afghanistan | Asia | Q3 | Group III |
| ACS 15 | PI 223817 | Afghanistan | Asia | Q1 | Group I |
| ACS 18 | PI 426218 | Afghanistan | Asia | Admixture | Admixture |
| ACS 19 | PI 426219 | Afghanistan | Asia | Q2 | Group II |
| ACS 20 | PI 278160 | Angola | Africa | Q3 | Group III |
| ACS 21 | PI 278161 | Angola | Africa | Q3 | Group III |
| ACS 22 | PI 254710 | Argentina | America | Q1 | Admixture |
| ACS 24 | PI 158045 | China | Asia | Q1 | Group I |
| ACS 26 | PI 158065 | China | Asia | Q1 | Admixture |
| ACS 28 | PI 162563 | China | Asia | Q1 | Admixture |
| ACS 29 | PI 195121 | China | Asia | Q1 | Admixture |
| ACS 32 | PI 436595 | China | Asia | Q1 | Group I |
| ACS 34 | PI 436598 | China | Asia | Q1 | Group I |
| ACS 36 | PI 436600 | China | Asia | Q1 | Group I |
| ACS 38 | PI 436603 | China | Asia | Q1 | Group I |
| ACS 40 | PI 532846 | China | Asia | Q2 | Group II |
| ACS 43 | PI 200108 | Egypt | Africa | Admixture | Admixture |
| ACS 46 | PI 298629 | Egypt | Africa | Q2 | Group II |
| ACS 49 | PI 238989 | Greece | Europe | Q2 | Group II |
| ACS 51 | PI 238991 | Greece | Europe | Q2 | Group II |
| ACS 60 | PI 156999 | India | Asia | Q1 | Group I |
| ACS 65 | PI 347847 | India | Asia | Q3 | Group III |
| ACS 68 | PI 257471 | India | Asia | Q3 | Admixture |
| ACS 70 | PI 222266 | Iran | Asia | Q2 | Group II |
| ACS 71 | PI 223014 | Iran | Asia | Q2 | Group II |
| ACS 72 | PI 223411 | Iran | Asia | Q3 | Group III |
| ACS 73 | PI 227253 | Iran | Asia | Q1 | Group I |
| ACS 74 | PI 229790 | Iran | Asia | Q2 | Group II |
| ACS 76 | PI 250748 | Iran | Asia | Q2 | Group II |
| ACS 80 | PI 250894 | Iran | Asia | Q1 | Admixture |
| ACS 81 | PI 250944 | Iran | Asia | Q2 | Group II |
| ACS 86 | PI 343821 | Iran | Asia | Q2 | Group II |
| ACS 87 | PI 343821 | Iran | Asia | Q2 | Group II |
| ACS 88 | PI 381030 | Iran | Asia | Q2 | Group II |
| ACS 91 | PI 198157 | Iraq | Asia | Q2 | Admixture |
| ACS 108 | PI 285170 | Israel | Asia | Q2 | Admixture |
| ACS 109 | PI 285171 | Israel | Asia | Q1 | Admixture |
| ACS 122 | PI 207667 | Japan | Asia | Q3 | Admixture |
| ACS 134 | PI 490031 | S. Korea | Asia | Q1 | Group I |
| ACS 137 | PI 490045 | S. Korea | Asia | Q1 | Group I |

**S1 Table.** Continued.

| **Accession No.** | **USDA**  **Genebank Entry / Cultivar Name** | **Country of origin** | **Continent** | **Cluster (Q) membership based  on STRUCTURE at K=3*** | **Group (G) membership based on PCoA analysis^¶^** |
| --- | --- | --- | --- | --- | --- |
| ACS 139 | PI 490046 | S. Korea | Asia | Q1 | Group I |
| ACS 141 | PI 490049 | S. Korea | Asia | Q1 | Group I |
| ACS 145 | PI 200107 | Myanmar | Asia | Q3 | Admixture |
| ACS 152 | PI 200427 | Pakistan | Asia | Q3 | Group III |
| ACS 153 | PI 250228 | Pakistan | Asia | Q2 | Admixture |
| ACS 155 | PI 292144 | Pakistan | Asia | Q3 | Group III |
| ACS 157 | PI 292147 | Pakistan | Asia | Q3 | Group III |
| ACS 158 | PI 292148 | Pakistan | Asia | Q3 | Group III |
| ACS 159 | PI 292149 | Pakistan | Asia | Q3 | Group III |
| ACS 163 | PI 426941 | Pakistan | Asia | Q3 | Group III |
| ACS 164 | PI 426942 | Pakistan | Asia | Q3 | Group III |
| ACS 168 | PI 426967 | Pakistan | Asia | Q3 | Group III |
| ACS 174 | PI 258369 | Russia | Asia | Q2 | Admixture |
| ACS 179 | PI 263461 | Russia | Asia | Q1 | Group I |
| ACS 181 | PI 263461 | Russia | Asia | Q1 | Group I |
| ACS 182 | PI 263465 | Russia | Asia | Q2 | Admixture |
| ACS 183 | PI 263469 | Russia | Asia | Q1 | Group I |
| ACS 185 | PI 265513 | Russia | Asia | Q2 | Group II |
| ACS 186 | PI 265517 | Russia | Asia | Q1 | Admixture |
| ACS 189 | PI 265522 | Russia | Asia | Q3 | Group III |
| ACS 191 | PI 269965 | Russia | Asia | Admixture | Admixture |
| ACS 193 | PI 254698 | S. America | America | Q1 | Admixture |
| ACS 194 | PI 254698 | S. America | America | Q2 | Admixture |
| ACS 195 | PI 200113 | Sri Lanka | Asia | Q3 | Admixture |
| ACS 197 | PI 253985 | Syria | Asia | Q2 | Group II |
| ACS 204 | PI 170710 | Turkey | Europe | Q3 | Admixture |
| ACS 215 | PI 170728 | Turkey | Europe | Q2 | Admixture |
| ACS 216 | PI 170729 | Turkey | Europe | Q2 | Group II |
| ACS 218 | PI 170732 | Turkey | Europe | Admixture | Admixture |
| ACS 220 | PI 170739 | Turkey | Europe | Admixture | Admixture |
| ACS 234 | PI 175907 | Turkey | Europe | Admixture | Admixture |
| ACS 241 | PI 179034 | Turkey | Europe | Q2 | Group II |
| ACS 242 | PI 179481 | Turkey | Europe | Q2 | Group II |
| ACS 246 | PI 179490 | Turkey | Europe | Q3 | Group III |
| ACS 253 | PI 238427 | Turkey | Europe | Q2 | Group II |
| ACS 278 | PI 240856 | Turkey | Europe | Q2 | Group II |
| ACS 285 | Landrace | Turkey | Europe | Q2 | Group II |
| ACS 304 | PI 238446 | Turkey | Europe | Q3 | Admixture |
| Ozberk-82 | Cultivar | Turkey | Europe | Q2 | Group II |
| Muganli-57 | Cultivar | Turkey | Europe | Q2 | Group II |
| ACS 325 | PI 254709 | USA | America | Q3 | Admixture |
| ACS 326 | PI 254709 | USA | America | Q1 | Admixture |
| ACS 329 | PI 280791 | USA | America | Q3 | Admixture |

**S1 Table.** Continued.

| **Accession No.** | **USDA**  **Genebank Entry / Cultivar Name** | **Country of origin** | **Continent** | **Cluster (Q) membership based  on STRUCTURE at K=3*** | **Group (G) membership based on PCoA analysis^¶^** |
| --- | --- | --- | --- | --- | --- |
| ACS 330 | PI 280791 | USA | America | Q1 | Group I |
| ACS 331 | PI 280794 | USA | America | Q3 | Group III |
| ACS 335 | PI 280812 | USA | America | Q1 | Admixture |
| ACS 337 | PI 542048 | USA | America | Q1 | Group I |
| ACS 344 | PI 599446 | USA | America | Q1 | Group I |
| ACS 348 | PI 599455 | USA | America | Q1 | Group I |
| ACS 353 | PI 599493 | USA | America | Q1 | Group I |
| ACS 356 | PI 153514 | Venezuela | America | Q3 | Admixture |
| ACS 358 | PI 254702 | Venezuela | America | Q1 | Admixture |
| ACS 359 | PI 320960 | Venezuela | America | Q1 | Admixture |

^*^ The cut-off probability for assignment to a cluster was 0.50 for clusters

^¶^ The cut-off probability for assignment to a cluster was 0.80 for grouping on STRUCTURE at *K*=3*
