# Supplementary material for: Genetic diversity and population structure of the Mediterranean sesame core collection with use of genome-wide SNPs developed by double digest RAD-Seq
Source: PLoS One. 2019 Oct 10;14(10):e0223757. doi: 10.1371/journal.pone.0223757 (PMC6786593; doi:10.1371/journal.pone.0223757)
Supplement: S2 Table — (DOCX) [file pone.0223757.s003.docx]

**S2 Table.** Genetic variation among four populations on diversity panel of 95 sesame accessions.

| **Populations (Origin of accessions)** |  | **Na^*^** | **Ne** | **I** | **Ho** | **He** | **PPL** |
| --- | --- | --- | --- | --- | --- | --- | --- |
| Asia | Mean | 2.033 | 1.509 | 0.477 | 0.082 | 0.310 | 99.66% |
|  | SE | 0.003 | 0.004 | 0.002 | 0.001 | 0.002 |  |
| Africa | Mean | 1.709 | 1.518 | 0.421 | 0.082 | 0.289 | 70.56% |
|  | SE | 0.006 | 0.005 | 0.004 | 0.002 | 0.003 |  |
| America | Mean | 1.925 | 1.465 | 0.435 | 0.134 | 0.283 | 91.61% |
|  | SE | 0.004 | 0.004 | 0.003 | 0.001 | 0.002 |  |
| Europe | Mean | 1.904 | 1.391 | 0.393 | 0.073 | 0.249 | 89.36% |
|  | SE | 0.005 | 0.004 | 0.003 | 0.001 | 0.002 |  |
| Grand mean | Mean | 1.893 | 1.470 | 0.431 | 0.093 | 0.283 | 87.80% |
|  | SE | 0.002 | 0.002 | 0.002 | 0.001 | 0.001 |  |

^*^Na, number of different alleles; Ne, number of effective alleles; I, Shannon diversity index; Ho, observed heterozygosity, He, expected heterozygosity and PPL, percentage of polymorphic loci
